# Primary Leiomyosarcoma of the Liver

**DOI:** 10.1155/1991/64252

**Published:** 1991

**Authors:** J. A. Paraskevopoulos, T. J. Stephenson, A. R. Dennison

**Affiliations:** 1 University Departments of Surgery Royal Hallamshire Hospital Sheffield S10 2JP UK; 2 University Departments of Surgery and Pathology Royal Hallamshire Hospital Sheffield S10 2JP UK; 3 Universität Bern Klinik für Viszerale und Transplantationschirurgie Switzerland; 4 University Surgical Unit Royal Hallamshire Hospital Sheffield S10 2JF UK

## Abstract

A case of primary leiomyosarcoma of the liver is described and the problems in diagnosis and management are discussed together with a review of the available literature. These tumours have a favourable prognosis when diagnosed preoperatively and the value of electron microscopic findings in this situation are highlighted.


*HPB Surgery*, 1991, Vol. 4, pp. 157–163
Reprints available directly from the publisher
Photocopying permitted by license only

© 1991 Harwood Academic Publishers GmbH
Printed in the United Kingdom

### CASE REPORT
### PRIMARY LEIOMYOSARCOMA OF THE LIVER

J.A. PARASKEVOPOULOS[1] and T.J. STEPHENSON[2]

*University Departments of Surgery[1] and Pathology[2], Royal Hallamshire Hospital, Sheffield S10 2JP, UK*

A.R. DENNISON

*Universität Bern, Klinik für Viszerale und Transplantationschirurgie, Switzerland*

*(Received 8 January 1991)*

A case of primary leiomyosarcoma of the liver is described and the problems in diagnosis and management are discussed together with a review of the available literature. These tumours have a favourable prognosis when diagnosed preoperatively and the value of electron microscopic findings in this situation are highlighted.

KEY WORDS: Liver tumours, leiomyosarcoma

### INTRODUCTION

Primary leiomyosarcoma of the liver is a recognised, but very rare, malignancy according to the World Health Organisation classification[1]. However, although these tumours are the least common primary mesenchymal malignancies at this site their relatively benign course, with slow progression and late metastasis, makes it important to establish the diagnosis preoperatively. A review of the available literature reveals only 21 previously described cases[2–8] and we have reviewed the diagnosis and treatment in the light of new imaging techniques and diagnostic modalities (particularly electron microscopic findings which are not previously described).

### CASE REPORT

A 62 year old man presented with a two year history of a painful mass in the right hypochondrium. The pain was constant, niggling in character, with no radiation and no exacerbating or relieving factors. His appetite had declined, resulting in a 6 kg weight loss over the same 2 year period. There were no other symptoms related to the gastro-intestinal tract. His medical history included intermittent claudication

Address correspondence to: J.A. Paraskevopoulos, M.D., F.R.C.S, University Surgical Unit, Royal Hallamshire Hospital, Sheffield S10 2JF, UK

(for which he underwent right aorto-iliac bypass) and per-anal removal of a rectal polyp.

On examination he looked unwell but was not anaemic or jaundiced. Abdominal examination revealed a large, spherical but smooth mass in the right hypochondrium which measured approximately 12 cm and was extending into the epigastrium. The mass moved downwards on deep respiration. There was no stigmata of liver disease.

Full blood count, urea, electrolytes and clotting screen were all normal. However, his liver function tests showed an alkaline phosphatase 130 u/l (normal range 35–105 u/l), a gamma GT 91 u/l (6–45 u/l) and an LDH 538 u/l (100–265 u/l). Trucut biopsies of the mass revealed a mesenchymal tumour of smooth muscle origin. Computerised tomograms of the abdomen showed a well demarcated swelling in the anterior part of the right lobe of the liver with no involvement of the inferior vena cava or portal vein (Figure 1). A visceral angiogram was performed using the Seldinger technique which confirmed that the mass was extending from the mid line across to the right lateral abdominal wall and also showed that the blood supply of the mass was predominantly from the right hepatic artery (with an additional small contribution from the left hepatic artery) (Figure 2).

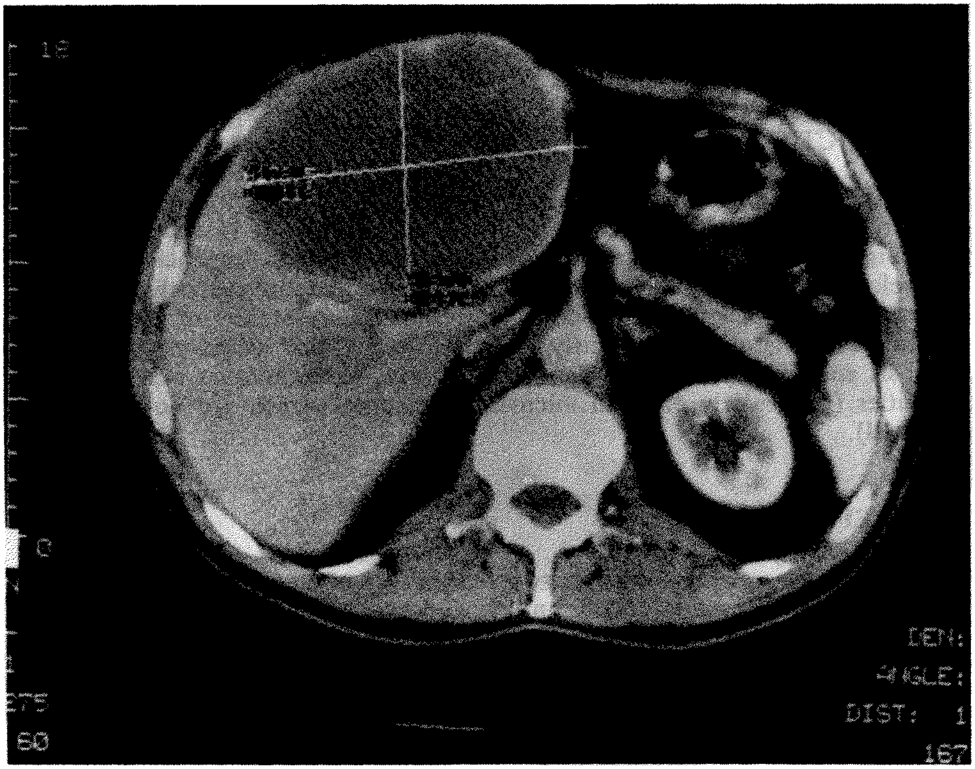

**Figure 1** Selected cut of CT liver scan. The large mass is located in the right lobe without involving the inferior vena cava.

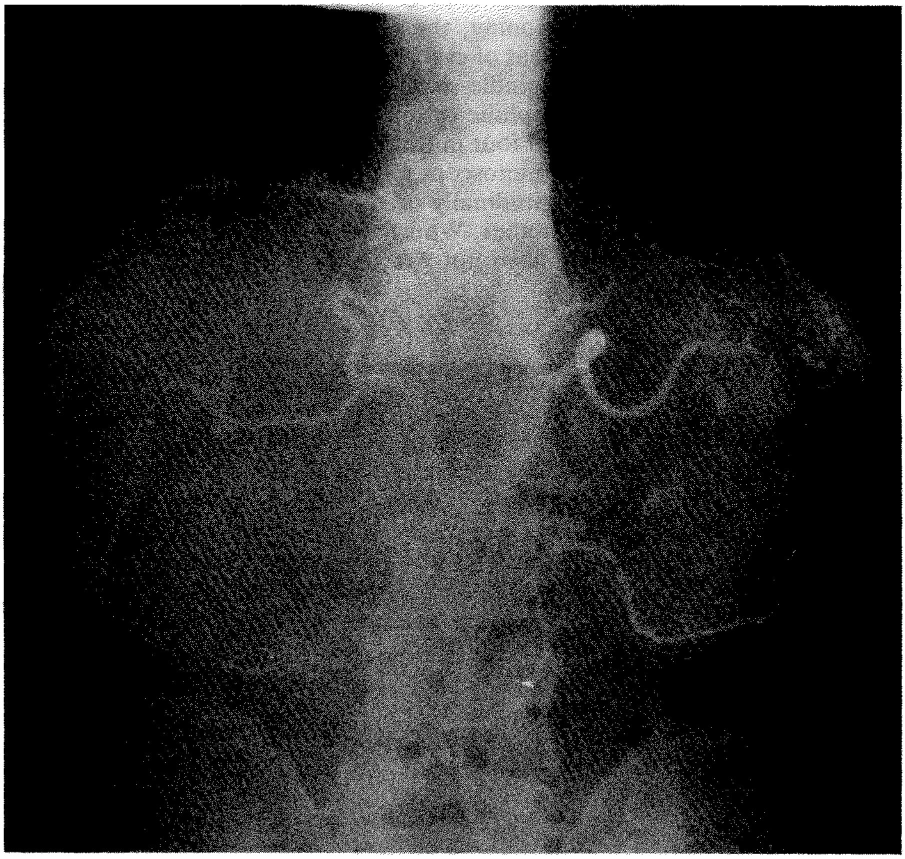

**Figure 2** Selective coeliac angiogram showing a large rounded mass with the main feeding artery originating from the right hepatic artery but with a small contribution from the left hepatic artery.

At operation a large tumour was found arising from the quadrate lobe of the liver. Laparotomy was otherwise normal with no evidence of other possible primary sites of leiomyosarcoma, and in particular, the stomach. The gallbladder was firmly adherent to the mass which was seen to be forcing the left and right lobes apart. There was no evidence of secondary spread or other intra-abdominal pathology and the mass appeared well encapsulated. The porta hepatis structures were also stretched widely by the expanding tumour but were separate from it, and it was possible to dissect the common duct and vessels and all the proximal left and right divisions from the mass with relative ease. The procedure was completed uneventfully and the patient remains alive and well five months post-operatively, with no sign of recurrence.

### HISTOPATHOLOGY

Naked eye examination showed a smooth surfaced cavitated round mass 14 × 13

× 11 cm with attached gallbladder 7 × 3 × 2.5 cm. The resection margin measured 15 × 6.5 cm. On sectioning, the mass was pale yellow coloured and apparently well circumscribed. Areas of cystic degeneration were present. Sections revealed interlacing fascicles of spindle-shaped cells containing elongated nuclei with blunt ends and having eosinophilic cytoplasm (Figure 3) typical of a smooth muscle tumour. Between three and four mitotic figures were visualised per 10 high power fields (Leitz Orthoplan microscope). Electron microscopy showed the cellular cytoplasm to contain longitudinally orientated 6 mm diameter placements (Figure 4) with associated dense bodies. Whilst these features are consistent with a low grade leiomyosarcoma they have not previously been described in this site. Excision appeared complete.

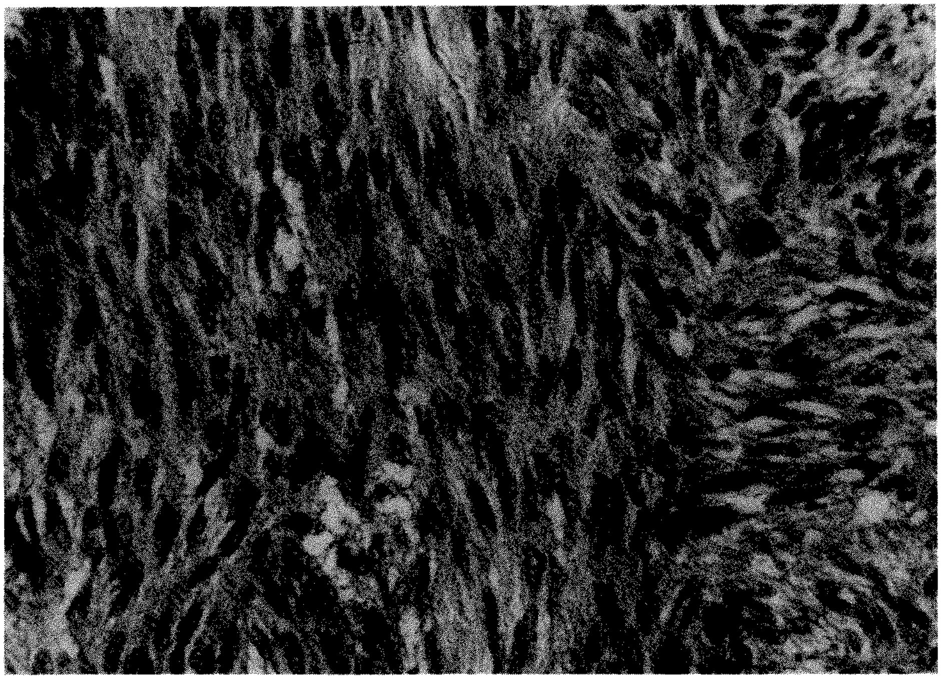

**Figure 3** The tumour comprises interlacing fascicles of spindle shaped cells with an elevated mitotic rate (H+E × 160).

### DISCUSSION

Primary liver malignancy is a relatively uncommon neoplasm in the western world, the overall incidence in one series of 19,967 autopsies performed over a 12 year period being 0.93%[9]. Primary mesenchymal tumours constitute only 1–2% of these primary malignant neoplasms of the liver, the presented case being the 22nd report of a primary leiomyosarcoma in the English literature.

The clinical presentation of this case is consistent with previous descriptions[5,7] but an important common feature of all the reported cases is the lack of specific

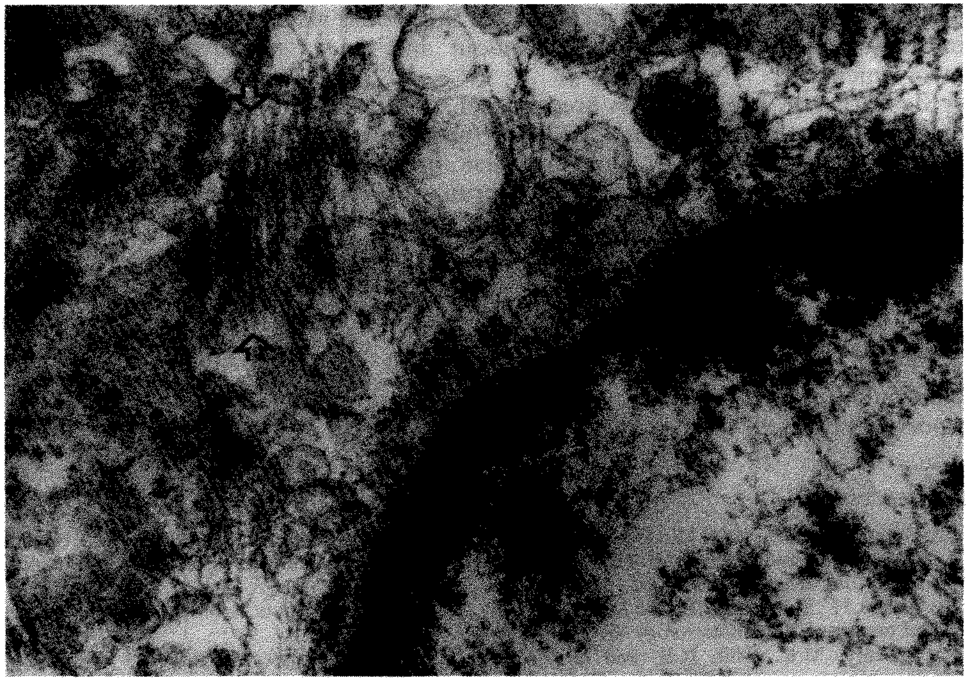

**Figure 4** Electron microscopy shows longitudinally arranged cytoplasmic 6 mm diameter filaments (arrowheads) with associated dense bodies. ( × 37500)

symptoms and the inconsistent, although frequent, derangement of the liver function tests (especially serum alkaline phosphatase, SGOT and serum lactic dehydrogenase). Although an abdominal mass with pain and weight loss is the common presentation, there is never sufficient evidence from the history, physical examination or laboratory data to suggest diagnosis of primary liver leiomyosarcoma. It is, however, important to consider this rare entity as the prognosis is much better than with the more commonly encountered hepatic malignancies (primary and secondary), particularly following surgical resection. The precise role of chemotherapy remains to be defined[4,7] but clearly this area is likely to present difficulties for some time due to the very small number of cases.

Accurate histology therefore remains the only method of diagnosing this entity pre-operatively with enough certainty to prevent inappropriate management. In the situation where uncertainty exists about the histological finding by routine methods, it is imperative to perform electron microscopy. It is very likely that this will provide valuable extra information at the least and with suitable biopsies will almost always confirm the diagnosis. In this respect, CT scan or ultrasound guided percutaneous liver biopsy is the method of choice although very occasionally percutaneous liver biopsy using the transjugular approach is regarded safer in patients with abnormal blood coagulation[10], although it requires considerable technical skill. These imaging methods combined with arteriography will delineate the anatomy of the mass and local vital structures and will help decide the operability when the histology becomes available. Recently peritoneoscopic biopsy and examination has been advocated[4] and this would also serve to give additional information about the size of the tumour and the extent of any extrahepatic involvement.

Mesenchymal liver tumours are unusual, especially the primary leiomyosarcomas. The fact that tissue diagnosis is the only available definite diagnostic investigation but the tumour runs a longer course, progresses more slowly and gives late metastases in comparison with other primary liver malignancies, renders preoperative biopsy vital. In view of this it seems reasonable to advocate routine preoperative biopsy of all solitary liver lesions. Subsequent surgical resection offers prolonged survival in the absence of disseminated disease.

*Acknowledgements*

We would like to thank Mr B. Fairbrother, Consultant Surgeon, for permission to report this case.

*References*

1. Goodman, Z.D. (1984) Histologic diagnosis of hepatic tumors. *Ann. Clin. Lab. Sci.*, **14**, 169–178
2. Iwatsuki, S. and Starzl, T.E. (1989) Experience with resection of primary hepatic malignancy. *Surg. Clin. North Am.*, **69**, 315--322
3. Masur, H., Sussman, E.B. and Molander, D.W. (1975) Primary hepatic leiomyosarcoma: a report of two cases. *Gastroenterology* **69**, 994–7
4. O'Leary, M.R., Hill, R.B. and Levine, R.A. (1982) Peritoneoscopic diagnosis of primary leiomyosarcoma of liver. *Human Pathol.*, **13**, 76–8
5. Chen, K.T.K. (1983) Hepatic leiomyosarcoma. *J. Surg. Oncol.*, **24**, 325–28
6. Qifang, P., Lunan, Y., Ruiti, R., Xianying, Y. (1987) Clinicopathologic manifestations in 2 cases of primary leiomyosarcoma of the liver. *Hua Hsi I Ko Ta Hsueh Hseuh Pao*, **18**, 90–2
7. Maki, H.S., Hubert, B.C., Sajjad,S.M., Kirchner, S.P. and Kuehner, M.E. (1987) Primary hepatic leiomyosarcoma. *Arch. Surg.*, **122**, 1193–6
8. Kinashita, A., Sakon, M. and Monden, M. *et al.* (1988) Triple synchronous malignant tumors. *Acta Chir. Scand.*, **154**, 477–9
9. Balazs, M. and Csermely, A. (1986) Primary tumours of the liver: a review of 249 cases. *Acta Morphol. Hung.*, **34**, 267–88
10. Adam, A. (1989) Percutaneous techniques in the liver and biliary system: recent advances. *Br. J. Hosp. Med.*, **42**, 102–10

*(Accepted by S. Bengmark 8 January 1991)*

### INVITED COMMENTARY

Primary hepatic leiomyosarcoma is extremely rare. It probably arises from hepatic vascular structures or bile ducts. Diagnosis of this tumour requires careful search to ascertain that it neither arises in an adjacent structure nor is metastatic from another primary. The mitotic index of five or more mitoses per 10HPF is the distinguishing factor between leiomyoma and leiomyosarcoma of uterine origin. This mitotic index, however, cannot predict the behavior of smooth muscle tumours of the gastrointestinal tract. The diagnosis of a low-grade hepatic leiomyosarcoma should be based on the tumour size, extensive areas of necrosis, mitotic rate of one to four mitoses per 10HPF, the presence of increased cellularity and lack of cellular anaplasia.

The clinical presentations and investigatory results are non-specific for the diagnosis of hepatic leiomyosarcoma. This tumour is slow growing and it has a much better prognosis compared with that of the more common hepatic malignant tumours. Wide surgical resection of the tumour is the treatment of choice for resectable lesions. Since symptoms from hepatic leiomyosarcoma arise mainly from compression of the parenchyma, palliative resection seems to be advisable even in the presence of distant metastases.

Professor Arthur K.C. Li
Department of Surgery
Prince of Wales Hospital
The Chinese University of Hong Kong
Shatin, N.T.
Hong Kong

### INVITED COMMENTARY

In addition to its specialized elements, the liver is composed of other various tissues and malignant tumors can arise from any of them. However, such mesenchymal tumors of the liver are uncommon. As authors indicate, primary leiomyosarcoma of the liver is extremely rare. This tumor is usually slow-growing and aggressive resection is useful even if metastases are present[1]. A correct preoperative diagnosis is not difficult with current imaging methods and biopsy technique. Against the author's description, there are several reports of electron microscopic findings of this tumor. According to Morales *et al.*[2], ultrastructural criteria of leiomyosarcoma is the presence of intracytoplasmic myofilaments; dense bodies in both cytoplasm and plasma membrane; pinocytic vesicles and invaginations of plasma membranes; and remnants of basal lamina or an excessive cell coat. The current case seems to fulfill such criteria.

### *References*

1. Masur, H. *et al.*, (1975) Primary hepatic leiomyosarcoma. *Gastroenterology*, **69**, 994
2. Morales, A.R. *et al.*, (1975) The ultrastructure of smooth muscle tumors with a consideration of the possible relationship of glomangiomas, hemangiopericytomas and cardiac myxomas. *Pathol. Annu.*, **10**, 65

Professor Naofumi Nagasue
Second Department of Surgery
Shiman Medical University
IZUMO 693
Japan

